# Lack of association between *PRNP *1368 polymorphism and Alzheimer's disease or vascular dementia

**DOI:** 10.1186/1471-2350-10-32

**Published:** 2009-04-08

**Authors:** Byung-Hoon Jeong, Kyung-Hee Lee, Yun-Jung Lee, Yun Joong Kim, Eun-Kyoung Choi, Young-Hoon Kim, Young-Sook Cho, Richard I Carp, Yong-Sun Kim

**Affiliations:** 1 Ilsong Institute of Life Science, Hallym University, 1605-4 Gwanyang-dong, Dongan-gu, Anyang, Gyeonggi-do 431-060, South Korea; 2 Samkwang Medical Laboratories, 9–60, Yangjae-dong, Seocho-gu, Seoul 137–887, South Korea; 3 New York State Institute for Basic Research in Developmental Disabilities, Staten Island, NY 10314, USA

## Abstract

**Background:**

Polymorphisms of the prion protein gene (*PRNP*) at codons 129 and 219 play an important role in the susceptibility to Creutzfeldt-Jakob disease (CJD), and might be associated with other neurodegenerative disorders. Several recent reports indicate that polymorphisms outside the coding region of *PRNP *modulate the expression of prion protein and are associated with sporadic CJD, although other studies failed to show an association. These reports involved the polymorphism *PRNP *1368 which is located upstream from *PRNP *exon 1. In a case-controlled protocol, we assessed the possible association between the *PRNP *1368 polymorphism and either Alzheimer's disease (AD) or vascular dementia (VaD).

**Methods:**

To investigate whether the *PRNP *1368 polymorphism is associated with the occurrence of AD or VaD in the Korean population, we compared the genotype, allele, and haplotype frequencies of the *PRNP *1368 polymorphism in 152 AD patients and 192 VaD patients with frequencies in 268 healthy Koreans.

**Results and conclusion:**

Significant differences in genotype, allele and haplotype frequencies of *PRNP *1368 polymorphism were not observed between AD and normal controls. There were no significant differences in the genotype and allele frequencies of the *PRNP *1368 polymorphism between Korean VaD patients and normal controls. However, in the haplotype analysis, haplotype Ht5 was significantly over-represented in Korean VaD patients. This was the first genetic association study of a polymorphism outside the coding region of *PRNP *in relation to AD and VaD.

## Background

Alzheimer's disease (AD), the most common cause of dementia in the aged population, is associated with progressive memory deterioration and disordered cognitive function resulting from a loss of cholinergic transmission and characterized neuropathologically by the presence of neurofibrillary tangles and amyloid plaques in the brain and clinically by gradual loss of memory. These changes may result from destructive processes involving the disruption of microtubule assembly and synaptic loss. Further neuronal damage and disease progression are consequences of this damage. Although the processes involved in AD could be triggered by many environmental factors, genetic studies have shown that in some cases mutations and polymorphisms of particular genes can confer susceptibility to the degenerative process. Several genes associated with AD have been identified, including amyloid precursor protein gene (*APP*), presenilin-1 gene (*PS1*), presenilin-2 gene (*PS2*), and the apolipoprotein E gene (*ApoE*) [1]. AD and prion diseases, such as Creutzfeldt-Jakob disease (CJD), share a number of clinical, pathogenetic and pathological features. A structural hallmark of AD is amyloid-β peptide (Aβ) aggregates in extracellular amyloid deposits defined as senile plaques, while in CJD there is an accumulation of abnormal protease-resistant isoform (PrP^res^) in neurons and in extracellular amyloid-like aggregates. Aβ-positive senile plaques in AD brains commonly contain PrP^C ^deposits [2-4] and incidental Aβ-positive senile plaques in prion diseases such as CJD may also be positive for PrP^C ^[5].

Vascular dementia (VaD) is the second most common cause of dementia after AD. VaD is a clinical syndrome causing cognitive decline due to cerebrovascular lesions. Risk factors for VaD are age, sex, race, hypertension, smoking, diabetes mellitus, and hypercholesterolemia. However, there is no conclusive evidence for the association of genetic polymorphisms with VaD. VaD and prion diseases share some pathophysiological similarities, such as the occurrence of dementia.

Prion protein contains 253 amino acids encoded by prion protein gene (*PRNP*), located on chromosome 20p12.3 in humans. *PRNP *plays an important role in conferring susceptibility or resistance to prion disease. A number of mutations in the open reading frame (ORF) are linked to the familial form of prion diseases [6,7]. Polymorphisms at codons 129 or 219 of *PRNP *are susceptibility factors to sporadic CJD [8-11]. In several European populations, an association between the *PRNP *codon 129 polymorphism and AD was reported [12-15]. In contrast to these studies, other studies failed to detect a significant association between this polymorphism and AD [16-18], and in Asian populations, no association between the *PRNP *codons 129/219 polymorphisms and AD was reported [19]. Recently, the polymorphism (*PRNP *1368) in an upstream of *PRNP *exon 1 was found to be associated with sporadic CJD in British and German populations [20,21], but this association was not seen in Dutch and Korean populations [22,23]. This polymorphism was studied in other diseases in addition to sporadic CJD. In a British population, there was no association of *PRNP *1368 polymorphism with frontotemporal lobar degeneration (FTLD) [24]. Although *PRNP *1368 polymorphism has been studied in sporadic CJD and FTLD patients, a case-controlled association study between the *PRNP *1368 polymorphism and either AD or VaD has not been reported thus far.

In the present study, the purpose was to investigate the genotype and allele frequency of a polymorphism outside the coding region of *PRNP *in Korean AD and VaD patients and to determine the correlation between this polymorphism and the incidence of AD and VaD in the Korean population.

## Methods

### Subjects

Analysis included 152 Korean patients with AD (51 male and 101 female; mean age at disease onset 73.48 ± 8.00 years), which were diagnosed according to the National Institute of Neurological and Communicative Disorders and Stroke-Alzheimer's Disease and Related Disorders Association (NINCDS-ADRDA) criteria [25] with minor modification specifying the gradual onset and progression of memory loss with a duration of at least 12 months. None of these patients reported family history of AD. All AD patients were gathered from Chunchon, South Korea and were examined in the Department of Neurology, Chunchon Sacred Heart Hospital. General medical and neurological examinations, neuropsychological testing, and computed topography scans were performed to exclude other forms of dementia. Blood samples were collected from 152 AD patients between May 2000 and June 2005. One hundred ninety two Korean patients with VaD (100 male and 92 female; mean age: 71.95 ± 8.92 years) were diagnosed according to the Diagnostic and Statistical Manual of Mental Disorders, Fourth Edition (DSM-IV) [26] and National Institute of Neurological Disorders and Stroke-Association Internationale pour la Recherche et l'Enseignement en Neurosciences (NINDS-AIREN) criteria [27] after clinical examination and neuropsychological testing, including mini-mental state examination (MMSE). VaD patients were gathered from Anyang, South Korea and were examined in the Department of Neurology, Hallym University Hospital. Blood samples were collected from 192 VaD patients between May 2000 and June 2008. The control subjects were 268 unrelated individuals (118 male and 150 female; mean age 71.17 ± 8.68 years) matched for age and ethnic background to AD patients and VaD patients (Table 1). All control subjects were volunteers recruited from routine health checkups at the Chunchon Sacred Heart Hospital. None of them presented symptoms of dementia or any movement disorders. Absence of dementia was determined by considering past history and Korean MMSE criterion (score of >24). Blood samples were collected from 268 healthy Korean volunteers between May 2000 and June 2005. The study was approved by the Ethical Committee of Chunchon Sacred Heart Hospital and an informed consent was given by all subjects or their caregivers. All blood samples were frozen at -70°C prior to analysis.

**Table 1:** Characteristics of AD and VaD patients and controls

	Control	AD	P value^a^	VaD	P value^a^
Number of subjects	268	152		192	
Gender					
Male, n (%)	118 (44.0%)	51 (33.55%)	0.014	100 (52.1%)	0.090
Female, n (%)	150 (56.0%)	101 (66.45%)		92 (47.9%)	
Mean age at disease onset (years ± SD)	-	73.48 ± 8.00	0.006	71.95 ± 8.92	0.348
Mean age at blood collection (years ± SD)	71.17 ± 8.68	-		-	

^a^Based on the difference between controls and AD or VaD patients

### Genotyping

Genomic DNA was extracted from 200 μl blood using the QIAamp DNA blood mini kit (Qiagen, USA) following the supplier's instructions. Polymerase chain reaction (PCR) was performed with J-1 (GAGAAAACCTTGCGTCAGCA) and J-2 (AAGGTGCAGAAAAGATGGGC) primers. These primers were designed to amplify a 586 bp product in an upstream region of *PRNP *exon 1. The PCR reagents contained 50 pmole of each primer, 5 μl of 10 × *Taq *DNA polymerase buffer, 1.5 mM MgCl_2_, 0.2 mM of each dNTP mixtures, and 2.5 units of *Taq *DNA polymerase (Promega, USA). The PCR conditions were 94°C for 2 min to denature, and 35 cycles of 94°C for 45 sec, 56°C for 45 sec, and 72°C for 1 min 30 sec, and then 1 cycle of 72°C for 10 min to extend the reaction. The Perkin-Elmer Cetus DNA thermal cycler (Pekin-Elmer, USA) was used. Restriction cleavage sites were searched using Webcutter, ver. 2.0 (Carolina Biological Supply Co., USA). A 20 μl aliquot of purified PCR mixture was digested at 37°C for 1 h with 5 units of *Pvu II *(Invitrogen, USA). Restriction products were separated on a 1.5% agarose gel and visualized with ethidium bromide staining under UV light. The purification of PCR products for sequencing was done using a QIAquick gel extraction kit (Qiagen, USA). The PCR products were directly sequenced on an ABI 377 automatic sequencer using a *Taq *dideoxy terminator cycle sequencing kit (ABI, USA) and the same primers as indicated earlier in the standard conditions.

### Statistical analyses

A χ^2 ^test was used to determine whether the *PRNP *1368 polymorphism was in Hardy-Weinberg equilibrium (HWE) in the Korean population. Odds ratios (OR) with 95% confidence interval (CI) and *P*-values were calculated by using the codominant model, controlling for age and sex as covariates. Differences in age of populations were analyzed using Student's *t*-test, and sex differences by using χ^2 ^test. Haplotypes and their frequencies were inferred using the algorithm developed by Stephens et al. [28]. Fisher's exact test was used to analyze differences in haplotype frequency between the normal population and patients with AD and VaD. The statistical powers were calculated using Statistical Power Calculator .

## Results

The genotype frequencies at *PRNP *1368 were in HWE in Korean control group (*P *= 0.742) and AD group (*P *= 0.226), not in VaD group (P = 0.025) (data not shown). To examine a correlation between the *PRNP *1368 polymorphism and susceptibility of AD in Koreans, we examined the genotype and allele frequencies of this polymorphism in 152 Korean AD patients and in 268 healthy controls. No significant difference between Korean AD patients and controls was found in genotype or allele frequency of the *PRNP *1368 polymorphism (Table 2). This result suggests that the *PRNP *1368 polymorphism does not increase susceptibility to AD. When our data set was stratified by gender, there was no significant association between this polymorphism and AD (data not shown).

**Table 2:** Genotype and allele frequencies of the PRNP 1368 polymorphism in the normal population, AD patients, and VaD patients

	Control (n = 268)	AD (n = 152)	VaD (n = 192)	AD *vs *Control			VaD *vs *control		
					
				OR^a^	95% CI^b^	P value	OR	95% CI	P value
Genotype frequency									
CC	103 (38.4)	56 (36.8)	84 (43.8)	-	-	-	-	-	-
CT	124 (46.3)	67 (44.1)	74 (38.5)	0.994	0.640 – 1.544	0.978	0.732	0.487 – 1.100	0.133
TT	41 (15.3)	29 (20.1)	34 (17.7)	1.301	0.731 – 2.315	0.371	1.017	0.594 – 1.742	0.952
Allele frequency									
C	330 (61.6)	179 (58.9)	242 (63.0)	-	-	-	-	-	-
T	206 (38.4)	125 (41.1)	142 (37.0)	1.119	0.839 – 1.491	0.444	0.940	0.717 – 1.232	0.654

Figures in parentheses are percentages

^a^Odds ratio

^b^Confidence interval

We also investigated the genotype and allele frequencies of *PRNP *1368 in 192 Korean VaD patients to determine whether this polymorphism correlated with VaD. There were no significant differences in genotype and allele frequencies between VaD patients and controls (Table 2). In addition, analysis of the haplotype frequency was performed in AD patients, VaD patients and controls. Six haplotypes of the 3 *PRNP *polymorphisms were constructed in Koreans. One (ht 5) of these six haplotypes was significantly over-represented in Korean VaD patients (Table 3).

**Table 3:** Haplotype frequency of three PRNP polymorphisms in the normal population, AD patients, and VaD patients

Haplotypes	1368	Codon 129	Codon 219	Frequency			P value		
				
				AD	VaD	Control	AD *vs *Control	VaD *vs *Control	AD *vs *VaD
ht1	C	A	G	0.5592	0.5622	0.5951	-	-	-
ht2	T	A	G	0.3618	0.3623	0.3450	0.479	0.451	0.996
ht3	T	A	A	0.0263	0.0142	0.0292	0.990	0.167	0.215
ht4	T	G	G	0.0197	0.0037	0.0115	0.280	0.252	0.050
ht5	C	A	A	0.0164	0.0352	0.0109	0.333	**0.013**	0.226
ht6	C	G	G	0.0066	0.0224	0.0083	1.0	0.078	0.197

## Discussion

In this study, we failed to detect a significant association between the *PRNP *1368 polymorphism and the occurrence of either AD or VaD in the Korean population.

There is the possibility that the *PRNP *1368 polymorphism is not functional with regard to affecting the level of PrP^C^. Another possibility is that a false negative result was obtained due to statistical powers. Data for AD and VaD patients showed a statistical power of 19.1% and 11.2%, respectively, at the Type I error rate of 0.05 compared with healthy controls. The statistical powers aren't high enough for ensuring that the *PRNP *1368 polymorphism is not relevant to prion replication. However, in the haplotype analysis among 3 *PRNP *polymorphisms, haplotype ht5 was the only haplotype significantly associated with VaD (p = 0.013) (Table 3) and the genotype frequency of *PRNP *1368 polymorphism in VaD patients was not in HWE. These results suggested some interaction among 3 *PRNP *polymorphisms in the determination of VaD risk and were needed for further evaluation of the association of *PRNP *1368 polymorphism with VaD in other ethnic groups.

Although the exact function of the prion protein is not fully understood, it might be involved in the development and intensity of oxidative stress and, thereby, contribute to neurodegeneration. Thus, polymorphisms in the coding region of *PRNP *might influence other neurodegenerative disorders in addition to prion diseases. Many studies on a correlation between the *PRNP *codon 129 and AD in various populations have yielded contradictory results [12-19]. This controversial result may be due to the different sample size of the population analyzed, to a difference in frequency of *PRNP *genotypes between different ethnic groups [29], or even to a difference in age of onset. In our previous studies, we failed to detect a significant association between *PRNP *polymorphism at codons 129/219 and the risk for AD or VaD in the Korean population [30,31].

Recently, there has been growing concern about several polymorphisms outside the ORF of *PRNP*, as there is evidence that levels of *PRNP *expression influence incubation time and the susceptibility to prion diseases. Polymorphisms in the *PRNP *promoter region may be associated with increased susceptibility of prion diseases in cattle and mice [32-34]. These *PRNP *promoter polymorphisms influence the *PRNP *gene expression level [35]. Overexpression of *PRNP *in transgenic mice led to a decrease in incubation time, whereas *PRNP *knockout mice were resistant to prion disease after infection [36,37]. Therefore, we suggest that the polymorphism of *PRNP *1368, located in the promoter region, may influence the expression of the *PRNP *gene; the promoter polymorphisms of *PRNP *might also be associated with other neurodegenerative diseases. In previous studies, several polymorphisms were identified in intronic and upstream regions of human *PRNP*. The single nucleotide polymorphism (SNP) at position -101 (*PRNP *12533) within the regulatory region of *PRNP *was associated with sporadic CJD in the British population [38], but not in samples derived from Dutch and German populations [20,39].

Even though these results did not show a relationship between *PRNP *1368 and AD or VaD, it would be useful to assess associations between AD/VaD and other *PRNP *polymorphisms in the promoter region, including the *PRNP *12533 polymorphism.

## Conclusion

*PRNP *1368 polymorphism was not significantly associated with incidence of sporadic AD and VaD in Koreans. However, in the haplotype analysis among 3 *PRNP *polymorphisms, we observed a significant association between haplotype ht5 and VaD. Our report is the first association study of a polymorphism outside the coding region of *PRNP *with AD and VaD.

## Competing interests

The authors declare that they have no competing interests.

## Authors' contributions

BHJ and YSK designed the study. BHJ, KHL, and YJL performed the genotyping. BHJ, YJK, EKC, YHK, YSC, and YSK analyzed the data. BHJ, EKC, and YJK performed statistical analysis. BHJ, YJK, RIC, and YSK wrote the paper. All authors read and approved the final manuscript.

## Pre-publication history

The pre-publication history for this paper can be accessed here:


